# Disinfection Strategies for Implant-Related Prosthetic Materials: An In Vitro Evaluation of Citric Acid, Chlorhexidine and Polyethylene Glycol

**DOI:** 10.3390/dj14010008

**Published:** 2025-12-22

**Authors:** Eduardo Escaf-Robles, Aritza Brizuela-Velasco, Daniel Robles-Cantero, Saray Fernández-Hernández, Javier Gil, Hector de Llanos-Lanchares

**Affiliations:** 1 Department of Surgery and Medical-Surgical Specialities, Oviedo University, 33003 Oviedo, Spain; eduardoescafrobles@outlook.es (E.E.-R.); llanoshector@uniovi.es (H.d.L.-L.); 2 Dens-IA Research Group, Faculty of Health Sciences, Miguel de Cervantes European University, 47012 Valladolid, Spain; abrizuela@uemc.es; 3 Faculty of Health Sciences, Miguel de Cervantes European University, 47012 Valladolid, Spain; sfernadezh@uemc.es; 4 Department of Materials Science and Engineering, Barcelona East School of Engineering EEBE, Universitat Politècnica de Catalunya, 08034 Barcelona, Spain; javier.gil.mur@upc.edu

**Keywords:** disinfection, prosthetic-components, citric acid, chlorhexidine, polyethylene glycol

## Abstract

**Background/Objectives**: There is evidence of possible contamination of prosthetic components originating from dental laboratories. The aim of this study is to investigate the disinfectant effect of citric acid and polyethylene glycol on implant-prosthetic materials in comparison with an untreated control and chlorhexidine. **Methods**: A total of 720 disks made of three different materials (titanium grade V, zirconia coated with feldspathic ceramic, and PMMA) contaminated with three bacteria (*Staphylococcus aureus*, *Enterococcus faecalis*, *and porphyromonas gingivalis*) were analyzed. Four treatment groups were tested: citric acid, polyethylene glycol, chlorhexidine and an untreated control group. Two assessment periods (3 and 21 days of incubation) were used, with bacterial metabolic activity measured using the resazurin reduction test and then analyzed by electron microscopy. **Results**: The results show that chlorhexidine has a superior inhibitory effect on all materials and bacterial strains in the short-term evaluation (3 days), while citric acid and polyethylene glycol showed higher efficacy after 21 days. Citric acid also exhibits differential effects when applied to grade V titanium. These differences were statistically significant at *p* < 0.05. **Conclusions**: There is evidence to recommend chlorhexidine for the disinfection of laboratory prosthetic components, but the enhanced effect of citric acid on grade V titanium and its long-term efficacy make it clinically promising candidate.

## 1. Introduction

Some studies have raised concerns about possible contamination of prosthetic components from dental laboratories. In this context, an in vitro study conducted in 2018 [1] analyzed the bacterial and fungal contamination of 202 implant abutments from 10 different dental laboratories. The results showed that *Micrococcus luteus*, *Bacillus subtilis*, *Staphylococcus epidermidis* and *Candida* were the dominant species.

This previously described situation may have clinical relevance, especially considering that the trialing of implant prostheses or even the placement of the final restoration may involve subgingival and occasionally subosseous positioning of laboratory-produced restorative materials. This placement can be particularly invasive for bone-level implants, as they lack a polished transmucosal collar, unlike tissue-level implants. An in vitro study by Teughels et al. [2] showed that contamination of implant-supported prosthetic components during the laboratory manufacturing process increases the likelihood of bacterial infiltration at the implant-abutment interface, which can lead to inflammation and peri-implant pathology. Their results showed that decontamination of the prosthetic components resulted in better soft tissue attachment around the implant and improved marginal bone level maintenance.

The materials used in implant-supported prostheses, many of which may come into contact with the subgingival connection described above, are very heterogeneous. They can be either prefabricated (categorized as Class IIb under the European Medical Device Regulation [MDR]) or custom-made. These materials include [3,4,5,6]:

- Grade V titanium (Ti_6_Al_4_V alloy), which is normally used for implant connections and metal frameworks.
- Zirconia, which, although not usually used directly at the implant interface due to its low shear strength, is often used as a framework or monolithic material.
- Polymethyl methacrylate (PMMA), which is used for temporary restorations or models for prosthetic trials.
- Feldspathic ceramics, typically used as a veneering material over structural substrates such as cobalt-chromium alloys, zirconia or titanium.

On the other hand, it is important to distinguish between sterilization, which eliminates all forms of microbial life—including bacteria, viruses, fungi and spores- and disinfection, which aims to significantly reduce the number of pathogenic microorganisms on inanimate objects. In this context, sterilization of prosthetic components from dental laboratories can be performed using methods such as steam (autoclaving) or argon plasma [7]. The former requires specific temperature, pressure and time conditions typically 121–134 °C at 1.1 to 2.1 bar to be effective. However, these conditions may alter the physical or chemical properties of the restorative materials. Argon plasma treatment usually requires relatively expensive equipment, which may limit its availability in routine clinical practice.

In contrast, disinfection with chlorhexidine is a method that is easy to perform in dental clinics. This antiseptic interacts with the bacterial cell membrane due to its positive charge and causes a change in membrane permeability and even destabilization through its effect on phospholipids. It also inhibits critical cellular functions such as protein synthesis and DNA replication, making it effective against a wide range of microorganisms, including Gram-positive and Gram-negative bacteria, fungi and certain viruses [8]. However, its effectiveness can vary depending on the type of microorganism and environmental conditions.

Another applicable method is ultrasonic cleaning, which is based on a generator that emits high-frequency ultrasonic waves (typically between 20 kHz and 40 kHz). These waves cause a cavitation phenomenon that is particularly suitable for cleaning hard-to-reach areas [9]. However, ultrasonic cleaning has traditionally been seen as a complementary method to disinfection or sterilization and not as a substitute.

Studies evaluating potential disinfection or sterilization methods for laboratory-produced components include both in vitro designs [9,10,11,12] and clinical studies [13,14,15]. In particular, in vitro studies are conducted with either implant abutments [10,12,16] or disks [11,17] made of different prosthetic materials. The disinfection methods most frequently compared in these studies include steam sterilization, argon plasma treatment, ultrasonic cleaning and- very often, even as a control, chlorhexidine.

However, the use of other chemical disinfection methods for laboratory-produced components, such as polyethylene glycol (PEG) or citric acid (CA), is less common. Clinically, both CA and PEG are inexpensive and widely available. CA is routinely accessible through pharmaceutical compounding and various dental applications, while PEG is an easily obtainable medical-grade polymer with a long history of use in healthcare and laboratory settings. Their cost and availability are comparable to those of chlorhexidine, and neither agent presents practical barriers to chairside preparation or application.

Glycols are chemical compounds formed by the reaction of water (two hydroxyl groups –OH) with ethylene oxide (C_2_H_4_O). Glycols are used in both vapor and liquid formulations due to their antimicrobial activity against a variety of microorganisms [18]. The cytotoxicity of PEG can vary depending on the length of the polymer chain (especially PEG 3350) and the concentration used [19]. Some authors have supported the use of PEG in various dental materials (e.g., orthodontic brackets) due to its potential effect in reducing bacterial adhesion [20]. Other in vitro studies in the field of endodontics have shown that PEG1000 has antibacterial activity against *Streptococcus mutans* and *Escherichia coli*, but only limited activity against other microorganisms, mainly *Enterococcus faecalis* [21].

Proposed mechanisms of PEG’s disinfectant activity include modification of surface wettability, reduction in microbial enzymatic activity and inhibition of microbial adhesion.

CA, in turn, is a tricarboxylic acid (C_6_H_8_O_7_) whose pH varies depending on its concentration in solution. In solid form, pure citric acid has a very low pH value (around 2 to 3), while in diluted solutions it can rise to pH values of around 4 to 6. CA is considered biocompatible, although it can have cytotoxic effects in high concentrations (above 1–2%). However, in certain dental applications -such as root canal irrigation in endodontics- CA at a concentration of 10% has not been shown to be cytotoxic, in contrast to 1% EDTA-T used as a control [22]. In dentistry, CA has been used for the disinfection of implant surfaces and showed effective results against *Porphyromonas gingivalis* in in vitro studies that were comparable to those of tetracyclines and superior to other chemical (EDTA) or physical (Nd:YAG laser) treatments [23]. CA is also commonly used in root canal irrigation protocols, occasionally in combination with other agents, as in the case of MTAD, which contains doxycycline, citric acid and sodium hypochlorite [24,25,26].

The mechanisms by which CA exerts its disinfectant effect include alteration of pH and denaturation of proteins, disruption of the bacterial cell membrane, reduction in biofilm formation, chelation of essential metal ions, influencing bacterial cell wall synthesis, and synergistic effects when used in combination with other disinfectants [22].

In summary, CA and PEG have demonstrated suitable antimicrobial potential and inhibition of bacterial adhesion in various biomedical contexts [18,19,20,21,22,23], including applications in the oral sphere [20,22,23,24,25,26]. Additionally, as previously noted, they are readily accessible, relatively low-cost products that can be easily implemented chairside. However, their potential use for disinfecting implant-supported prosthetic components from the dental laboratory has been scarcely explored, providing the basis and rationale for their inclusion in this study.

The aim of this in vitro study was to assess the antibacterial and disinfectant potential of polyethylene glycol (PEG) and citric acid (CA) on contaminated prosthetic materials by measuring bacterial metabolic activity and to compare their efficacy with that of chlorhexidine and an untreated control group.

## 2. Materials and Methods

### 2.1. Materials Used for the Samples

A total of 720 disks with a diameter of 5 mm and a thickness of 2 mm were used as specimens, which were made of three different materials commonly used in implant-supported prosthetic rehabilitation: Grade V titanium (Ti), polymethyl methacrylate (PMMA) and zirconia (Zr). All three materials were treated to reflect their typical clinical surface texture: Titanium and PMMA were polished, while the zirconia disks were glazed with a 20 µm thick layer of feldspathic ceramic.

The sample size was based on previous studies with comparable objectives and design, in which smaller sample sizes (n = 3–5) were used [11,17]. The present n (10) was therefore considered sufficient to allow for reproducibility and to identify consistent biological trends while adhering to the principles of experimental refinement and reduction in accordance with current ethical standards for in vitro research.

### 2.2. Bacterial Strains

The bacterial strains used in the experiment were *Staphylococcus aureus (S. aureus)* (CECT, 8753), *Enterococcus faecalis (E. faecalis)* (CECT, 4102) and *Porphyromonas gingivalis (P. gingivalis)* (ATCC, 33277). We selected *Staphylococcus aureus* and *Enterococcus faecalis* due to their well-known roles as opportunistic pathogens in prosthetic and nosocomial infections. *Porphyromonas gingivalis* was included as a representative periodontal/peri-implant pathogen of high clinical relevance. It is one of the core bacteria associated with peri-implantitis and periodontal disease, with virulence factors such as fimbriae and gingipains that facilitate adhesion, invasion, and disruption of host tissues [27]. Since *P. gingivalis* is a strict anaerobic organism, a container was used during the test to generate an anaerobic atmosphere (GasPak EZ Anaerobic Container System Sachets with Indicator, BD, Franklin Lakes, NJ, USA).

Todd-Hewitt Broth (THB) (Oxoid, Basingstoke, UK, Ref. CM0335) was used as the culture medium for the growth of *S. aureus* and *E. faecalis*, while *P. gingivalis* was cultured in Tryptic Soy Broth (TSB) (Oxoid, Basingstoke, UK, Ref. CM0127). Both are enriched media to promote bacterial growth. Sterile Milli-Q water, previously autoclaved under standard conditions, was used to dissolve 36.4 g of THB and 30 g of TSB powder per liter.

Before sowing, the disks were immersed in 70% pharmaceutical ethanol for 10 min for sterilization. Subsequently, all procedures were performed under a laminar flow hood (Telstar Bio II Advance Plus, Azbil Telstar Technologies, Barcelona, Spain) to ensure a sterile environment and avoid contamination.

For bacterial seeding, the inoculum was prepared in such a way that a similar bacterial count was achieved for all strains. First, an optical density of 0.2 was achieved using an optical density meter (Biochrom Ultrospec 10, Cambridge, UK). Two serial dilutions, 1:10 and 1:100, were then performed and used for seeding.

### 2.3. Disinfection Methods

The solutions of CA (40%) and PEG (10%) were prepared in the laboratory using monohydrated citric acid (Sigma-Aldrich Corp, St. Louis, MO, USA, CAS 5949-29-1) and polyethylene glycol 6000 (PEG 6000) in synthesis grade (Merck KGaA, CAS 25322-68-3, Darmstadt, Germany), respectively. Similarly, 0.2% chlorhexidine (Clorhexidina Lacer^®^, Laboratorios Lacer, Barcelona, Spain) was used, as a method commonly used in clinical practice, but also in studies with similar design and objectives. The disinfectants were applied for different exposure times, according to the protocols reported in previous studies [28,29,30]: 30 s for citric acid and chlorhexidine and 60 s for polyethylene glycol. Finally, a control group without disinfectant treatment was included for all tested materials and the three bacterial strains.

Regarding the disinfection protocols with CA and PEG, the selected concentrations and exposure times were based on previously published studies. Although some authors have used lower CA concentrations (e.g., 12.5%) [23], their protocols involved substantially longer application times (up to 5 min). In our study, a higher concentration (40%) was chosen within the range commonly reported in dental research (30–50%) [25] but applied for a shorter duration to minimize the risk of surface alterations in the tested materials.

For PEG, the selected 10% concentration is supported by evidence showing that PEG400–6000 exhibits bacteriostatic activity, anti-adhesive behavior, and enzymatic inhibition at concentrations between 5% and 15% [20,21]. However, PEG requires a longer exposure time to modify surface hydrophilicity and interfere with bacterial activity; therefore, a 60 s application was used, consistent with protocols reported in the literature [20,21].

The evaluation of bacterial metabolic activity and the electron microscopic examination of the samples were carried out after 3 days and the procedure was repeated after 21 days. Table 1 shows the relationship between materials, disinfection methods and evaluation times.

### 2.4. Experimental Test

#### 2.4.1. Assessment of Bacterial Metabolic Activity

A reagent called resazurin (resazurin sodium salt, Sigma-Aldrich, Darmstadt, Germany, Ref. R7017), at a concentration of 5 mg/mL, was used to measure the metabolic activity of the bacteria seeded on the samples, and the procedure was repeated identically at two time points after seeding: 3 and 21 days. Resazurin, with the chemical name sodium-7-hydroxy-3H-phenoxazin-3-one-10-oxide, is a redox indicator that changes color according to the metabolic activity of the bacteria. In its oxidized form it is blue, but when the bacteria are metabolically active, they reduce it to resorufin, which is pink. The higher the metabolic activity, the more intense the pink color and consequently the greater the bacterial viability.

Once the solution was prepared, the culture medium was removed from the samples, they were transferred to a new plate, and resazurin was applied, keeping them in an incubator at 37 °C for 1 h. Absorbance was measured using the TECAN Infinite 200 PRO microplate reader (Tecan Group Ltd., Männedorf, Switzerland) at two specific wavelengths: 570 nm and 600 nm. These measurements allowed recording of the optical signal corresponding to the metabolic activity resulting from the reduction in the resazurin reagent. The i-control 2.0 software (Tecan Austria GmbH, Grödig, Austria) was used, which directly exports the quantitative results of the observance to an Excel 2024 (Microsoft Corp., Redmond, WA, USA) spreadsheet for subsequent data analysis.

#### 2.4.2. Calculation of the Correlation Factor (R_0_)

This factor corrects the difference in absorbance between both wavelengths in the absence of bacterial activity.

$$R_{0}=\frac{A_{570}}{A_{600}}$$

#### 2.4.3. Percentage of Resazurin Reduced

Once R_0_ has been calculated, it is used to determine the proportion of resazurin reduced by cellular activity in each sample.

$$A_{R_{red}}=\left[A_{570}-(A_{600}\times R_{0})\right]\times 100$$

#### 2.4.4. Electron Microscopy Evaluation of Treated Surfaces

Sample preparation for electron microscopy included prior fixation of the bacteria with 2.5% glutaraldehyde and subsequent dehydration the following day by immersion in ethanol of various concentrations (20–40–60–80–96 and 100%) for 15 min at each concentration.

Immediately prior to observing the samples under the microscope, additional preparation was required if the samples were not electrically conductive, as was the case with the feldspathic coated zirconia and PMMA samples. These samples had to be coated with carbon using the Leica EM ACE600 sputter coater (Leica Microsystems GmbH, Wetzlar, Germany).

A program called “Pulse Double—CT” was selected, which is optimized for double-pulse carbon coating. The material used for vaporization was carbon (CT) and the coating was applied with a thickness of 10.0 nm. The operating conditions included a working distance of 50 mm, a sample tilt of −20° and no rotation. The process was performed under a vacuum pressure of 1.0 × 10^−4^ mbar to ensure homogeneous and high-purity deposition. The images were taken with an accelerating voltage of 20 kV and a working distance (WD) of approximately 10 mm, as this had to be adjusted in order to focus and observe the surface correctly depending on the sample.

#### 2.4.5. Statistical Analysis

Data were analyzed using statistical software (Minitab release 13.0; Minitab Inc., State College, PA, USA). The normal distribution of the data was verified using the Shapiro–Wilk test. As the assumptions of normality and homoscedasticity were satisfied, a 2-way analysis of variance (ANOVA) was performed to assess the effect of the disinfectant agent and material type on the percentage of resazurin reduction for each incubation period. Post hoc pairwise comparisons were carried out using the Tukey test when significant differences were detected. The level of significance was set at *p* < 0.05. Each experimental condition was tested in 10 specimens (n = 10), resulting in a total of 720 disks analyzed.

## 3. Results

### 3.1. Assessment of Bacterial Metabolic Activity at 3 and 21 Days of Evaluation

**Figure 1:**
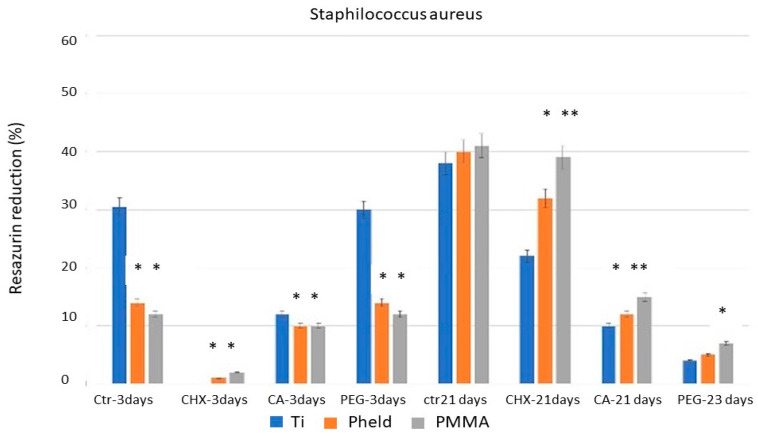
Results of the percentage resazurin reduction test on Titanium Grade V, Zirconia coated with feldspathic ceramic and PMMA surfaces, colonized with *Staphylococcus aureus* after 3 and 21 days of evaluation. Asterisks indicate statistically significant differences between materials (*p* < 0.05).

**Figure 2:**
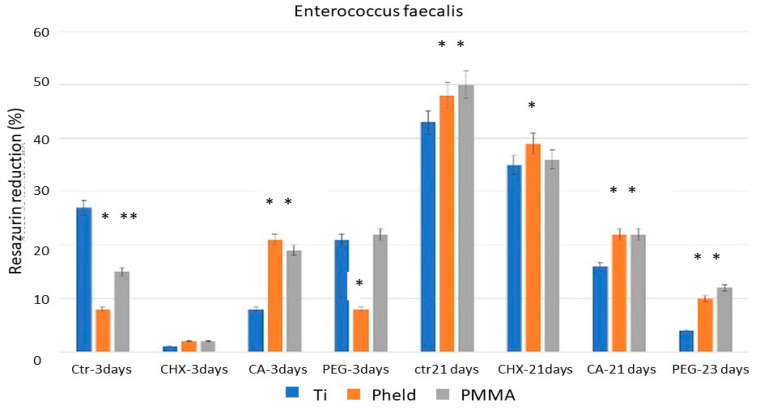
Results of the percentage resazurin reduction test on Titanium Grade V, Zirconia coated with feldspathic ceramic and PMMA surfaces, colonized with *Enterococcus faecalis* after 3 and 21 days of evaluation. Asterisks indicate statistically significant differences between materials (*p* < 0.05).

**Figure 3:**
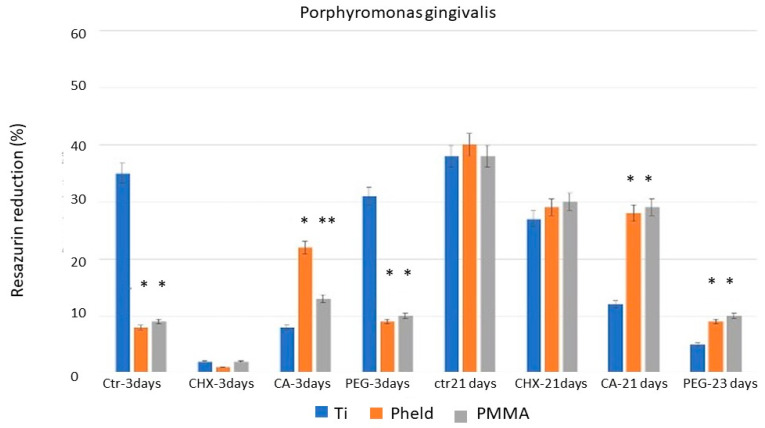
Results of the percentage resazurin reduction test on Titanium Grade V, Zirconia coated with feldspathic ceramic and PMMA surfaces, colonized with Porphyromonas gingivalis after 3 and 21 days of evaluation. Asterisks indicate statistically significant differences between materials (*p* < 0.05).

Figure 1, Figure 2 and Figure 3 show the percentage of resazurin reduction, grouped by the tested bacterial strains, in blocks corresponding to the different disinfectant treatments analyzed at the two evaluation periods (3 and 21 days). Likewise, the condensed significance matrix (Table 2) presents the statistical significance (*p* < 0.05) between materials (grade V titanium, feldspathic-coated zirconia, and PMMA) according to treatment and incubation period.

For all three bacteria, at 3 days of evaluation period, the titanium samples showed a statistically significant decrease (*p* < 0.05) in the percentage of reduced resazurin with chlorhexidine (CHX) and citric acid (CA) treatments compared with untreated samples, i.e., the positive control. This indicates that metabolic activity and, consequently, bacterial viability were clearly affected and reduced by the chlorhexidine (CHX) and citric acid (CA) treatments, though to a lesser extent with citric acid. However, treatment with polyethylene glycol (PEG) did not reduce the percentage of reduced resazurin. Similarly, for all three bacteria at 3 days of evaluation period, the results for zirconia coated with feldspathic ceramic and PMMA were very similar. In both cases, a reduction in the resazurin reduction percentage was observed after treatment with chlorhexidine (CHX), but citric acid (CA) and polyethylene glycol (PEG) treatments did not produce a decrease, indicating that bacterial viability was not significantly affected, even compared with the same samples under control treatment.

**Figure 4:**
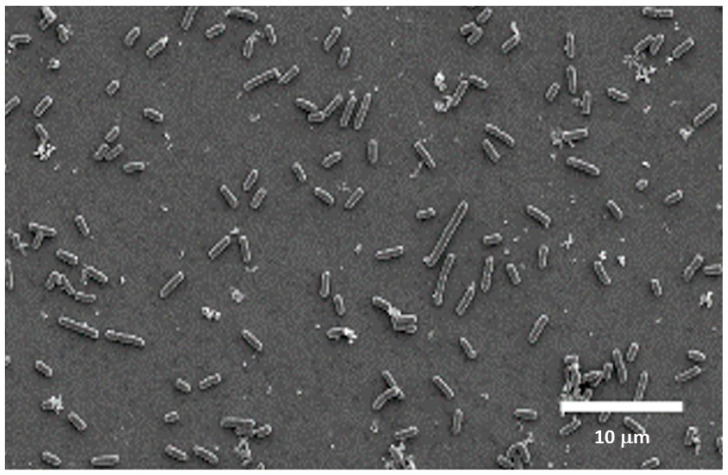
Image of *P. gingivalis* on a titanium disk treated with polyethylene glycol (PEG) after 21 days of incubation.

At 21 days of evaluation period, the first notable finding is that in the control treatments, for all three bacteria and all three tested materials, a similar reduction in the percentage of resazurin is maintained, ranging from slightly below 40% to 50%. The results regarding the decrease in bacterial metabolic activity show significant differences after 21 days of incubation compared with those obtained after 3 days, as described in Figure 1, Figure 2 and Figure 3. Essentially, the effect of chlorhexidine decreases with a longer evaluation period (21 days), and the test results become similar to those of the control samples. However, samples treated with citric acid maintained a stable bactericidal effect similar to that observed at the shorter evaluation time (3 days), particularly on grade V titanium. The most remarkable finding concerns polyethylene glycol; due to its bacteriostatic property, no bacterial death was observed under the scanning electron microscope, but very few bacteria were detected on the surface (Figure 4).

### 3.2. Electron Microscopy Evaluation of Treated Surfaces at 3 Days of Follow-Up

**Figure 5:**
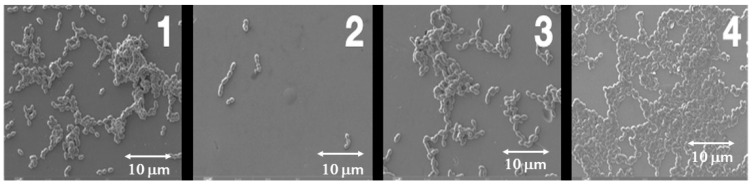
Representative SEM image of PMMA disks seeded with *P. gingivalis* under different treatment conditions: (1) untreated control, (2) CHX, (3) CA, and (4) PEG after 3 days of follow up.

Overall, the results of the electron microscopic evaluation of the surfaces of the different materials, disinfection treatments, and bacterial strains support the metabolic activity test results described above using resazurin reduction. A trend is confirmed regarding the differential efficacy of citric acid depending on the material, with a clear ability to reduce the activity of the three bacterial strains studied (*S*. *aureus*, *E*. *faecalis*, and *P*. *gingivalis*) on grade V titanium. However, the SEM study also confirms the ineffectiveness of PEG and the limited effect of CA on the other two materials tested (zirconia coated with feldspathic ceramic and PMMA) for all three bacteria, as shown in Figure 5 for PMMA.

## 4. Discussion

The aim of the present experimental in vitro study was to determine the efficacy of the application of CA or PEG treatments on commonly used prosthetic materials for implant-supported restorations as a disinfectant against three specific bacterial strains and to compare it with both an untreated control and a clinically common and easy-to-use method such as CHX.

The results of the bacterial metabolic activity tests, corroborated by SEM images, showed that CHX was the treatment with the greatest disinfecting capacity after 3 days of incubation. These results may be consistent with those reported in certain clinical studies [14,15], in which disinfection of prostheses with CHX prior to their final insertion was associated with less peri-implant marginal bone loss during the follow-up period.

CA, in turn, showed variable efficacy depending on both the material to which it was applied and the time of evaluation. For all three strains, the results on feldspathic ceramic and PMMA-coated zirconia after 3 days of incubation did not differ from those obtained with PEG or the untreated control samples. However, a significant difference was observed when applied to grade V titanium. This variability suggests a material-dependent interaction between the chemical treatment and the titanium surface, probably related to the ability of citric acid to alter bacterial adhesion conditions on titanium, but not on materials with lower ionic affinity or different surface charges, and even to the possible formation of oxidizing compounds that affect bacterial viability [23]. Furthermore, when the test was repeated after 21 days of incubation, CA showed a stronger inhibitory effect on bacterial metabolic activity than CHX. Taken together, these two circumstances suggest that CA may have real potential for the disinfection of laboratory-fabricated grade V titanium components, a material that is frequently used in clinically critical areas from a contamination point of view, such as at the implant-abutment connection and in transgingival or even subosseous sites, which is much more common than with the other two materials tested (feldspathic ceramic- coated zirconia and PMMA).

Finally, PEG does not act through membrane disruption or immediate bactericidal mechanisms but instead exerts a progressive bacteriostatic and anti-adhesive effect. Peng et al. [20] demonstrated that PEG-modified surfaces exhibit significantly reduced bacterial attachment, an effect attributed to increased hydrophilicity and steric repulsion at the interface. Nalawade et al. [21] also reported inhibitory effects of PEG solutions in the 5–15% range, associated with reduced enzymatic activity and impaired cell–surface interactions rather than direct cell killing. These mechanisms help explain the pattern observed in the present study: the limited effect at 3 days, consistent with the absence of immediate bactericidal action, followed by a clear inhibitory response at 21 days, supported by SEM evidence showing reduced bacterial colonization on PEG-treated samples.

Although the use of both CA and PEG is clinically simple and economically feasible, they have rarely been considered as alternatives for prosthesis disinfection. In vitro studies [11,17] with disk models similar to ours have tested different disinfection methods such as ultrasound or steam and occasionally others with high economic costs (laser, argon plasma or photofunctionalization).

On the other hand, the bacteria used in the present study are consistent with those used in some studies with a similar design and objective [17,23], but for reasons of manageability or availability it was not possible to use other pathogens commonly observed in the contamination of prostheses, such as *Micrococcus luteus, Bacillus subtilis, Staphylococcus epidermidis* and *Candida* [1].

Similarly, the results were analyzed for materials commonly used in prosthetic rehabilitation over implants, although for reasons of practicability, other materials that would also have been of interest were not considered—such as cobalt-chromium, lithium disilicate or even composites. In addition, zirconia was used, although the measurements were carried out on the side of the disk with the most commonly used finish in prosthetics, namely feldspathic ceramic glaze. Either of these two materials would have been of interest on their own.

Nevertheless, our results as well as previous in vitro studies on abutments [10,12] and clinical reports [14,15] indicate that disinfection by various physical or chemical means should be clinically encouraged, as a reduction in bacterial activity on contaminated materials and even a trend towards preservation of peri-implant marginal bone at follow-up of up to 60 months has been observed [13].

It must be acknowledged that this study is not without limitations. The combined management of several independent variables (material–bacterium–disinfection method–follow-up period) significantly increased the number of procedures and samples required (720), but at the same time resulted in a limited number of n per combination (10). However, this corresponds to the sample sizes used in studies with similar designs [11,17] subject to the same difficulties.

Finally, the results of this study open up new research directions that may be of interest in the future. The differential effect of CA on grade V titanium and its sustained activity over prolonged incubation periods are remarkable in themselves but could be enhanced if one considers that several studies have shown a tendency for improved adhesion of fibroblasts to titanium previously treated with CA [31]. Thus, treatment of prostheses with CA would not only provide the benefit of disinfecting effect but would also promote peri-implant soft tissue adhesion to some extent in the early stages after loading of the prosthesis. In addition, future investigations, such as the study by Mehl and coworkers [11], should also evaluate the potential structural or physicochemical changes that CA and PEG treatments may induce in different prosthetic materials- for example, in terms of surface roughness (which may affect per se bacterial adhesion) or wettability.

## 5. Conclusions

Within the limitations of this in vitro study, the following conclusions can be drawn:

Chlorhexidine (CHX) showed the strongest short-term disinfecting effect on all materials and bacterial strains tested, supporting its continued use as a clinically reliable agent for prosthesis disinfection.

Citric acid (CA) showed a persistent and material-dependent bactericidal effect, particularly on grade V titanium, where its efficacy was evident at both 3 and 21 days. These results suggest that CA could be a clinically relevant alternative for the disinfection of titanium prosthetic components, particularly at the implant-abutment interface.

Polyethylene glycol (PEG) did not show a direct bactericidal effect; however, its progressive bacteriostatic trend after 21 days suggests a potential to limit bacterial adhesion that warrants further investigation as a complementary approach.

## Figures and Tables

**Table 1:** Each experimental block described above—resulting from the combination of materials, disinfectant agents, and evaluation times (240 units)—was reproduced for each of the three bacterial strains under study (*S. aureus*, *E. faecalis*, and *P. gingivalis*), yielding a total of 720 disks analyzed.

Disk Material	3 Days of Incubation				21 Days of Incubation			
	Untreated Control	CHX	CA	PEG	Untreated Control	CHX	CA	PEG
Grade V Titanium	10	10	10	10	10	10	10	10
Zirconia coated with feldspathic ceramic	10	10	10	10	10	10	10	10
PMMA	10	10	10	10	10	10	10	10

**Table 2:** Condensed significance matrix. * = Statistically significant differences (*p* < 0.05); ✖ = Not significant.

Bacteria Test Time	Control	CHX	CA	PEG
*S. aureus*—3 d	* Ti ≠ Zr, PMMA	* Ti ≠ Zr, PMMA	* Ti ≠ Zr, PMMA	* Ti ≠ Zr, PMMA
*S. aureus*—21 d	✖	* Ti ≠ Zr, PMMA + Zr ≠ PMMA	* Ti ≠ Zr, PMMA + Zr ≠ PMMA	✖
*E. faecalis*—3 d	* Ti ≠ Zr, PMMA + Zr ≠ PMMA	✖	* Ti ≠ Zr, PMMA	* Ti ≠ Zr + Zr ≠ PMMA
*E. faecalis*—21 d	* Ti ≠ Zr, PMMA	* Ti ≠ Zr + Zr ≠ PMMA	* Ti ≠ Zr, PMMA	* Ti ≠ Zr, PMMA
*P. gingivalis*—3 d	* Ti ≠ Zr, PMMA	✖	* Ti ≠ Zr, PMMA + Zr ≠ PMMA	* Ti ≠ Zr, PMMA
*P. gingivalis*—21 d	* Ti ≠ Zr	✖	* Ti ≠ Zr, PMMA	* Ti ≠ Zr, PMMA

## Data Availability

The original contributions presented in this study are included in the article. Further inquiries can be directed to the corresponding author.

## Abbreviations

The following abbreviations are used in this manuscript:

CA	Citric Acid
CHX	Chlorhexidine
DNA	Deoxyribonucleic Acid
EDTA	Ethylenediaminetetraacetic Acid
MDR	Medical Device Regulation
PMMA	Polymethyl methacrylate
PEG	Polyethylene Glycol
Ref	Reference
SEM	Scanning Electron Microscope
THB	Todd-Hewitt Broth
Ti	Titanium
Zr	Zirconia
UK	United Kingdom
USA	United States of America
WD	Working Distance
